# Improving Efficiency of Passive RFID Tag Anti-Collision Protocol Using Dynamic Frame Adjustment and Optimal Splitting

**DOI:** 10.3390/s18041185

**Published:** 2018-04-12

**Authors:** Muhammad Qasim Memon, Jingsha He, Mirza Ammar Yasir, Aasma Memon

**Affiliations:** 1 Faculty of Information Technology & Beijing Engineering Research Center for IoT Software and Systems, Beijing University of Technology, Beijing 100124, China; memon_kasim@yahoo.com; 2 Institute of Information & Communication Technology, Mehran University of Engineering & Technology, Jamshoro 76062, Pakistan; mirzaammaryasir86@gmail.com; 3 School of Economics and Management, Beijing University of Technology, Beijing 100124, China; kaasma.bjut@gmail.com

**Keywords:** radio frequency identification, RFID anti-collision, RFID tags, radio transceiver, wireless sensor network, dynamic BTSA, optimal splitting

## Abstract

Radio frequency identification is a wireless communication technology, which enables data gathering and identifies recognition from any tagged object. The number of collisions produced during wireless communication would lead to a variety of problems including unwanted number of iterations and reader-induced idle slots, computational complexity in terms of estimation as well as recognition of the number of tags. In this work, dynamic frame adjustment and optimal splitting are employed together in the proposed algorithm. In the dynamic frame adjustment method, the length of frames is based on the quantity of tags to yield optimal efficiency. The optimal splitting method is conceived with smaller duration of idle slots using an optimal value for splitting level $M_{opt}$, where (M > 2), to vary slot sizes to get the minimal identification time for the idle slots. The application of the proposed algorithm offers the advantages of not going for the cumbersome estimation of the quantity of tags incurred and the size (number) of tags has no effect on its performance efficiency. Our experiment results show that using the proposed algorithm, the efficiency curve remains constant as the number of tags varies from 50 to 450, resulting in an overall theoretical gain in the efficiency of 0.032 compared to system efficiency of 0.441 and thus outperforming both dynamic binary tree slotted ALOHA (DBTSA) and binary splitting protocols.

## 1. Introduction

**Figure 1:**
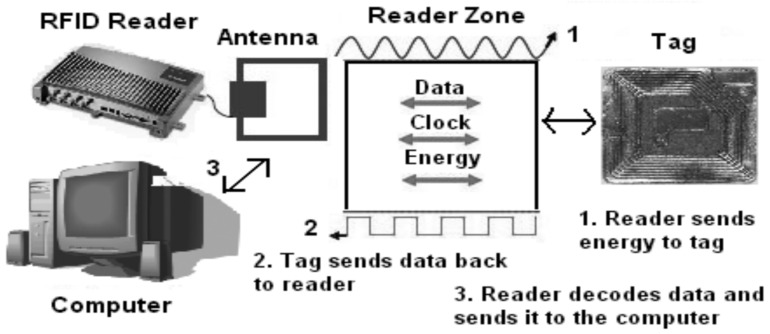
Components and process of the radio frequency identification (RFID) system.

RFID is a low power and low-cost wireless technology, which enables tracking and identification of objects such as credit cards for access applications, tracking of shipping containers, anti-theft objects in stores and tags used as screw-shapes to recognize wooden items [1,2,3]. The key function of RFID is to store, send and process information between a tag and a reader. However, RFID application is not without issues and the key issue is the RFID signal collision problem that can be resolved by improving the efficiency of tag collection. Figure 1 shows RFID system components including tag or transponder, transceiver or reader or interrogator, antenna, and the host computer. Literature suggests that a variety of RFID anti-collision protocols have been developed, such as tree-based approach [1,4], ALOHA-based approach [5,6] and tree-ALOHA-based approach [7]. These approaches mostly follow the Q algorithm and the Binary Tree (BT) algorithm. In ALOHA-based anti-collision protocols including ALOHA, slotted ALOHA, and frame slotted ALOHA, tags are scheduled to be transmitted at distinct time to lower the probability of tag collisions [8,9].

Electronic Product Code (EPC) global Class-1 Gen.2, also known as the Q algorithm, is based on frame slotted ALOHA [8,10] in which the frame size depends on the parameter Q. The frame length is initially adjusted in relation to tag quantity for optimized efficiency. In order to obtain optimal efficiency, the number of tags should be estimated. Conventional estimate methods [11,12] such as Vogt estimate [6], maximum a posteriori estimate [13] and Bayesian estimate [14] are not exact estimates as they do not explore the optimized quantity of tags. In addition, the higher the quantity of tags, the higher the estimation time as well as the degree of computational complexity. On the other hand, conventional estimate methods are generally less efficient and produce inaccurate calculations when the frame length exceeds the total quantity of tags with the number of slots, either idle or successful, as being zero [15,16]. ALOHA-based protocols, such as enhanced dynamic frame slotted ALOHA (EDFSA) [17] and tree slotted ALOHA (TSA) [18], separate tags into groups and interrogate each group at the same time, thus reducing the probability of collisions and improving the performance efficiency in the range of 34–37%. In the TSA protocol, tag estimation is carried out via inducting various reading cycles by adding new nodes in the tree considering that the collision has occurred in the slot. This implies that the process becomes redundant in the case of non-occurrence of collision in the cycle. In RFID technology, a number of collisions are produced during wireless communication between the reader and a number of tags, raising various issues, such as the number of iterations required in the analysis of the collided tags, the number of idle slots, the complexity of computation, identification time, wastage of bandwidth, and consumption of energy. In RFID, the reader usually interrupts a slot in the case of no response from tags for a pre-defined time duration, resulting in a short idle slot with the size of which remaining constant [19].

In our proposed algorithm, a probabilistic dynamic adjustment approach is followed in compensating the length of the frame, which is based upon observing the primary slot rather than each slot in the Q algorithm. The length of the frame can be close to the quantity of tags. When it exceeds the quantity of tags, however, the probability of the initial slot being idle also increases. Alternatively, in the case that the frame length does not exceed the quantity of tags, the probability of a collision for the initial slot increases [10,20].

One objective of this research is to observe whether idle slots with a small size using optimal splitting would produce better results compared to binary splitting. We then propose an anti-collision protocol corresponding to optimal splitting (optimal level as M) to replace binary splitting.

Our proposed algorithm has the following main features:

- We utilize dynamic frame adjustment and optimal splitting in the proposed algorithm (DFA-OS) to improve the efficiency performances of the passive RFID tag anti-collision protocol.
- The dynamic frame adjustment method would adjust the length of a frame to make it closer to a value that corresponds to the quantity of tags to yield optimal efficiency, which is better than existing protocols, such as dynamic BTSA (DBTSA).
- The proposed algorithm is a non-estimation-based protocol, meaning that the efficiency of the protocol is constant when the quantity of tags increases.
- The optimal splitting method is conceived with a smaller time duration for idle slots, making it outperform binary splitting protocol.
- Dynamic frame adjustment and optimal splitting (DFA-OS) are applied together in a way that is different from existing non-estimation-based protocols that have been proposed based on improved frame adjustment method, such as DBTSA, in conjunction with the optimal splitting scheme.
- The efficiency of the proposed algorithm is achieved through reducing the number of idle slots as well as identification time compared to existing protocols, such as a DBTSA, adoptive BTSA and splitting BTSA that only proposed to reduce the number of collisions.

The rest of the paper is organized as follows. Section 2 contains the related work. Section 3 briefly elaborates on conventional anti-collision algorithms such as the BTSA algorithm and M-ary splitting algorithm. Section 4 describes the proposed algorithm. Section 5 presents simulation results. Finally, concluding remarks are drawn in Section 6.

## 2. Related Work

In RFID, two major concerns are emphasized: RFID tag collisions and RFID missing-tag events. The first issue requires that the emphasis be placed on reducing the number of collisions while the second one on scanning a group of known tags and identifying missing tags in the current round [21,22]. When the number of tags is equal to the number of slots in the initial frame, BTSA achieves the highest efficiency value of 0.43 [23], which is greater than the pure binary tree protocol [24] and the dynamic frame slotted ALOHA (Dynamic FSA) [12] with the same efficiency as the tree slotted ALOHA protocol (TSA) [18,25]. Different protocols have applied BTSA, such as Modified Q [26], estimation based FSA and binary selection [27] and adoptive binary splitting (ABS) [28]. Adoptive binary splitting that is carried out without estimation would not improve efficiency in case the number of tags varies. Enhanced dynamic FSA (EDFSA) [17] and modified Q algorithm perform estimation to obtain optimal efficiency. However, such estimation cause some other issues such as high computational complexity and diversity of the number of tags, producing results of varying efficiency and failing to introduce the estimation error to improve efficiency.

Protocols based on ALOHA decrease the probability of tag collisions by disseminating tag responses progressively [12]. These protocols treat tags at different slots by separating the access time of tags. In slotted ALOHA, tags respond prior to time slots [10,12,21], whereas in frame slotted ALOHA (FSA), time slots are further grouped into frames inside which tags respond [29,30]. In the ALOHA based protocols, identification efficiency is based on the quantity of tags as well as the number of slots, whose performance may be lowered when the quantity of tags is less than the number of slots, resulting in idle slots, or when the quantity of tags is more than number of slots, inducing collision occurrence. Therefore, in order to estimate the number of slots compensated with tags, protocols based on ALOHA are mostly applied [6,13,14,17]. However, these protocols are not deterministic and need to be well related to the number of tags, therefore, estimation should be conducted. Dynamic FSA [6,13,17,31,32] adjusts frame length with consecutive frames, which may include slots, to set up the identification process. Dynamic FSA has been merged with tree algorithms [15,18,23,26,33] to improve identification efficiency.

Non-estimation protocols based on ALOHA are also known as Q algorithms [34], which either compensate frame size by considering each slot or require estimation before adjustment of the frame size [34,35]. Various improved Q algorithms have been proposed [36,37] to enhance the efficiency through changing step size without involving estimation. However, hybrid protocols [38] could improve the performance in enhancing efficiency and outperform improved Q algorithms.

## 3. Preliminaries

### 3.1. Conventional Algorithm

BTSA combines splitting algorithms by incorporating binary tree (BinTree) with slotted ALOHA. BTSA compensates the length of frame dynamically, and thus is known as the dynamic BTSA (DBTSA) algorithm [39]. In DBTSA, frame length adjustment is similar to the Q algorithm except that it adjusts the length by judging the introductory or initial slot, rather than each and every slot. The DBTSA algorithm consists of different functions, such as function BTSA Length(L), which is responsible for adjusting frame length. When the reader activates the command and sends it to the tag for data sharing purposes with $L=2^{Q}$, the tag receives a query adjustment and randomly allocates a number ranging from $0\;to\;2^{Q}-1$. The reader receives a tag response and identifies a collision in the frame, and the length of the frame varies increasingly when the collision occurs by invoking function Q=min(15,Q+1). When the tag response is idle, the frame is compensated by function Q=max(0,Q−1). If tag response is successful, then the frame is adjusted with function Q=Q+0. To compensate for the frame length, collision occurs by the tag response in the initial slots. Therefore, the frame adjustment procedure of DBTSA expediency is beyond the usual strategy to decide frame length adjustment based on primitive slots; thereby, the reader can receive a moderate and justified frame length. As collision occurs at the tags, they are eradicated through the binary search tree rule using BinTree, achieving the primary idea of a binary tree. However, when the collision occurs, the inexpediency of binary search tree is transpired inside the slots. This may result in the production of a number of iterations while eradicating collided tags; therein, the identification time increases and the efficiency of anti-collision algorithms is reduced.

### 3.2. Dynamic BTSA (DBTSA)

For DBTSA algorithm in comparison to the ALOHA algorithm, the main difference is determined by the total quantity of tags and in adjusting the length of frame. In addition, when the frame length is higher than the quantity of tags, the number of idle (empty) slots (by identifying only introductory slot) will increase. However, if the length is smaller than the quantity of tags, the number of collisional slots (by identifying only introductory slot) will increase, meaning that the efficiency of the given algorithm will degrade in both scenarios. DBTSA algorithm comprises of dynamic frame adjustment and the binary tree algorithm, which do not conduct estimation. In DBTSA, dynamic frame adjustment considers the compensation of the initial frame length using innovation of ALOHA and frame adjustment method to assimilate to the Q algorithm [40] used in the proposed frame adjustment method. The Q algorithm is a non-estimation-based algorithm with an efficiency that is higher than some of the estimation based algorithms [17]. DFSA and hybrid algorithms have the expediency over the Q algorithm due to the frame adjustment procedure as previously pointed out [20,40,41].

**Figure 2:**
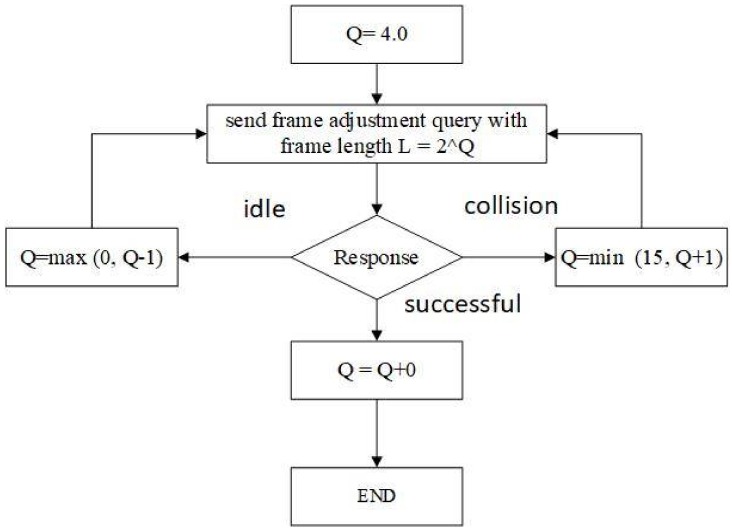
Dynamic frame adjustment in the proposed algorithm.

In Figure 2, the frame adjustment method of the proposed algorithm is described. The frame is divided into a number of slots which is randomly chosen by the tag, initially with Q=4 and length $L=2^{Q}$. When the reader receives a response from tag, if the response indicates a collision occurring in the primary slot, the reader rounds Q=min(15,Q+1) and invokes an anti-collision algorithm to resolve the collided tags (in the initial slot). The reader also broadcasts a command with new length to identify responses produced by tags in the primitive slot of the consecutive frames. If the tag response appears to be successful, the reader rounds Q=max(0,Q−1) and the operation moves towards the next step with Q unchanged. If the first slot is idle, rendering no responses received from the tag, the reader rounds Q=max(0,Q−1) and broadcasts a new message L, thus identifying new tag responses.

### 3.3. Performance of Existing Tag Anti-Collision Schemes

**Figure 3:**
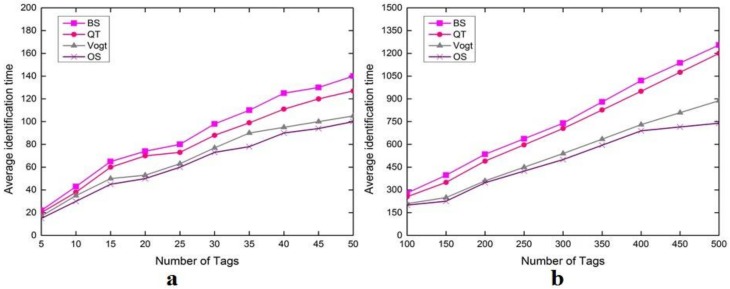
Comparison of the average identification time of four anti-collision schemes: BS, QT, Vogt and OS. (**a**) Number of tags in the range 5–50; (**b**) number of tags in the range 100–500.

Figure 3 shows the performance of four tag anti-collision schemes, i.e., binary splitting (BS), query tree (QT), ALOHA-based scheme (Vogt), and optimal splitting (OS), respectively. The results show the average identification time by Monte Carlo method with different numbers of tags ranging from 0 to 50 (Figure 3a) and from 100 to 500 (Figure 3b). This performance evaluation is performed based upon some assumptions. First, slot size ratio is set to 0.1. Second, when the frame size is equal to the number of tags, the Vogt’s estimations are evaluated, while the estimation time of these tags is not measured due to identification delay [42], since the time is very large; thus performance difference is less comparable to the OS scheme. Third, tags’ IDs in QT are orderly distributed. Fourth, tags are static within a specific identification round while being dynamic between identification rounds, since the tags are considered to move with low speed for dynamic objects or even static objects. In Figure 3, BT and QT identification time is longer than OS and even longer than the Vogt ALOHA-based scheme, which can be inferred that in OS identification, time is less important than all the schemes. Therefore, OS outperforms all the schemes and transpired better average identification, thereby, producing less collisions and idle slots than Vogt’s ALOHA-based scheme.

## 4. The Proposed DAF-OS Algorithm

### 4.1. The $M_{opt}$ Splitting Algorithm

In RFID, an idle slot is terminated by the reader sooner than other types of slots due to null response from tags for a pre-defined duration of time, resulting in a shorter idle slot. The $M_{opt}$ splitting algorithm is an estimation-less algorithm, where M is chosen carefully with M > 2, which is the best choice over binary splitting protocol. The selection of an optimal value for M renders a perfect standard estimation of M that varies over the slot measure [19]. Slot size is usually constant in a given RFID system and the optimal value for M is usually determined offline with less identification time. Due to smaller size of idle slot and thus short recognition time, the splitting level generates much better performance. In this algorithm, every tag is identified with counter C and number Q, which is generalized within {0,1,…,M−1}. Tags labelled with C=0 have n chances to respond whereas tags labelled with C>0 react only when given the opportunity. Tags labelled with C<0 are those that have already been recognized, and thus stop responding during the current query round. The reader broadcasts a message with r=0, 1, 2 to indicate different actions such as collision, successful, and idle event. Responded tags arbitrarily produce Q and fetch C with Q. On receiving a collision, the remaining tags would increase C by M−1. All the tags are divided into several M individual groups by using a splitting level with the optimal value.

**Figure 4:**
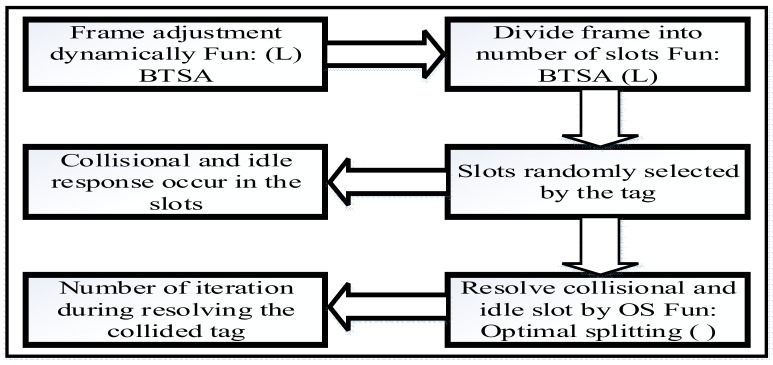
Process of the proposed DAF-OS algorithm.

The process of the proposed algorithm is shown in Figure 4 that is comprised of the frame adjustment length$=2^{Q}$, where {Q=0,1,2,3,…,n}. The RFID reader communicates with the tag in a specific sensing area, adjusting specific slot length in a frame as well as splitting the frame into various numbers of slots and going through query adjustment with a reasonable frame length L. The tag counter randomly selects an integer ranging from 0 to L−1 and randomly selects the slots. When the counter is set to zero, the reader detects the tag reaction in the slot as it receives the tag ID. When the tag response in the initial slot occurs as collisional, the reader moves the command of length L and tag to find primitive consecutive frame in the initial slot. If the tag response is idle, the reader moves a command with new length and detects the response from the tag in the slot. When the reader receives the collisional response by the tag in the slot, the reader invokes an anti-collision algorithm to resolve the collided tags in the slot. While resolving, the remaining tags are put on hold until the collided tags are completely recognized. In addition, the number of iterations are produced and the iteration counter calculates the number of iterations. When the initial slot of the frame receives successful response from the tag, Q remains unchanged and the reader moves to the optimal splitting scheme. Here, two functions are incorporated in the DFA-OS algorithm. First, function DBTSA (L) is implemented for dynamic frame length adjustment. Second, optimal splitting function OS (L) is implemented to resolve the colliding/idle slots.

**Figure 5:**
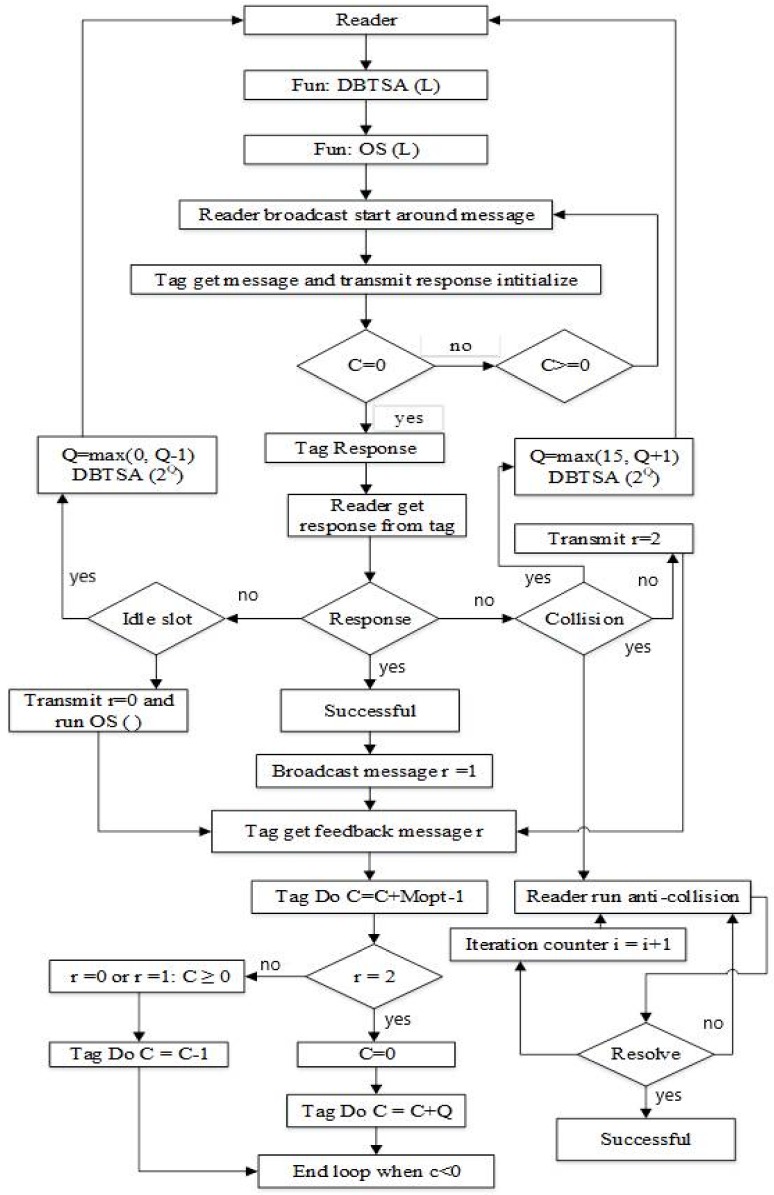
Flowchart of the proposed DAF-OS algorithm.

Figure 5 is the flow chart of the proposed DFA-OS algorithm in which function DBTSA(L) and OS(L) are employed to resolve tag collision. If a tag is successfully recognized, the reader sends a message with r=1 to the tag. The tag receives the message from the reader to indicate that the tag has been recognized.

### 4.2. Fun: OS(L)

In the OS algorithm, during each iteration of tag recognition, an integer counter C and an arbitrary number generator Q assigned with a value within {0, 1……M−1} are maintained. When the value of M is low, tags are divided into one or more groups with at least one tag in each group. Therefore, the number of idle slots tends to be high, resulting in longer identification time. However, as the value of M goes beyond a certain value, the chance of collision starts to increase, also causing identification time to increase. Since it is true that the size of an idle or incomplete slot is always smaller than that of a collision or successful slot, when the value of M increases, identification time would first increase and then decease. In the algorithm, when collision occurs, the reader sends a message with r=2 to the tags. Upon receiving the message, the collided tags are divided into $M_{opt}$ groups where ($M_{opt}$ > 2). For tags with C=0, set C=C+Q where Q is selected from [$0,1,\ldots ,M_{opt}-1$] and for tags (C > 0), set $C=C+M_{opt}-1$. The pseudo codes of proposed DFA-OS algorithm for the reader and for the tag are described in Algorithm 1 and Algorithm 2, respectively.
**Algorithm 1. Pseudo code of DFA-OS:** reader side 1   Q = 4.0 2   Dynamic BTSA $\left(2^{Q}\right)$ function Dynamic BTSA $\left(2^{Q}\right)$ 3   send adjustment command with $2^{Q}$ 4   get answer of transponder 5   if transponder collision 6    then Q=min(15,Q+1) and Dy: $2^{Q}$ 7   else if idle answer 8    Q=max(0,Q−1) and Dy: $2^{Q}$ 9   else if successful answer  10    BTSA $\left(2^{Q}\right)$   function BTSA (L)  11   transmit command with Length and c = 0  12   do {take transponder answer and find collision        if transponder collision  13    Send feedback = collision and perform Fun: OS() }  14   else if no answer  15    send feedback = idle  16   else transponder answer  17    take ID by transponder and reply = successful   18   end   19   C=c+1 while c<L   function OS (L)  20   Initial transmit active message  21   take answer by transponder   22   loop if reply collisional   23   transmit r=2 and run algorithm  24   else if iteration  25     then transmit r=−1 and run algorithm  26   if reply idle  27     then transmit r=0  28   else if successful   29     then transmit r=1  30   end loop   31   when no more answer   32   transmit inactive message
**Algorithm 2. Pseudo code of DFA-OS:** tag side 1   starting counter c = 0 2   while set command with $2^{Q}$ 3   do {c = 0 to $2^{Q}-1$ 4      if c = 0 send id end } 5   if take command with $2^{Q}$ 6   c = 0 to $2^{Q}-1$ 7   while c ≥ 0 8   do {c = transponder find (counter) } function id () 9   starting count c = 0  10   loop when c ≥ 0   11   (c = 0) transponder answer  12   transponder receive feedback message r  13   do $C:=C+\;M_{opt}-1$  14      if r = 0 or 1: (c ≥ 0) transponder   15   do c:c−1  16      if r = 2 (c = 0) transponder  17   do c = c + q  18   else do i = i + 1 when iteration  19   end loop when c < 0

The identification time approaches to its optimum as the size of idle slot becomes smaller. The size of each slot (successful, collision or idle) is determined by the optimal value of M, which only requires information about the number of initial slots. OS(L) uses the optimal splitting (OS) algorithm to derive the optimal splitting level M. The major advantage of the OS algorithm is apparent since it produces a smaller number of iterations to resolve tag collision, resulting in shorter identification time and thus higher efficiency of the RFID system [43].

### 4.3. Fun: DBTSA(L)

The reader uses BTSA(L) to adjust the length of messages that it sends to the tags where $L=2^{Q}$. The counter value is a random integer number ranging from $0\;to\;2^{Q-1}$. If the reader receives a tag response and recognizes a collision in the frame, the length is updated through re-computing Q=min(15,Q+1). If there is an idle slot, the length is also updated through re-computing Q=max(0,Q−1). When the response is successful, the length is not changed.

## 5. Experiment and Analysis

The proposed algorithm (DFA-OS) has been implemented in the MATLAB environment with the main parameters of the simulation being taken from the EPC Class1 Gen. 2 protocol. Evaluation of DAF-OS in the experiment has included the following metrics: number of iterations, idle slots and collisions as well as amount of identification time. Comparison of the efficiency of DAF-OS with DBTSA algorithm was also performed.

**Figure 6:**
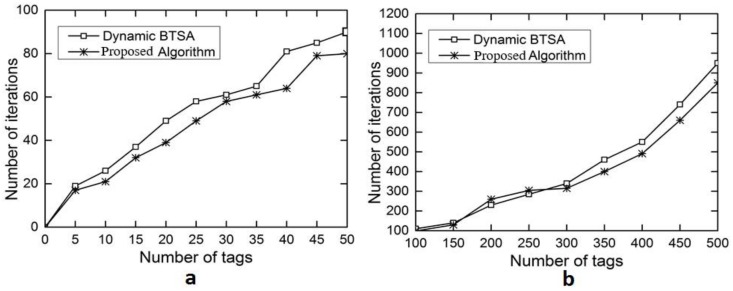
Comparison on the number of iterations between DAF-OS and DBTSA. (**a**) Number of tags in the range 5–50; (**b**) number of tags in the range 100–500.

**Figure 7:**
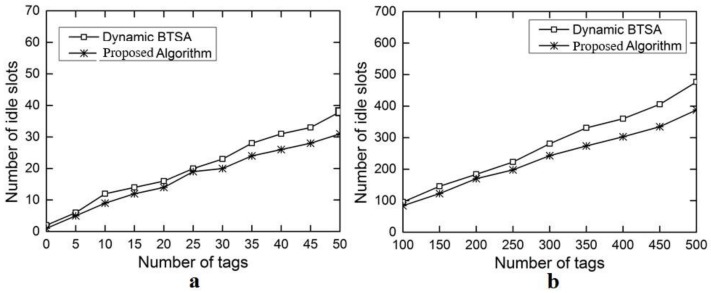
Comparison on the number of idle slots between DAF-OS and DBTSA. (**a**) Number of tags in the range 5–50; (**b**) number of tags in the range 100–500.

**Figure 8:**
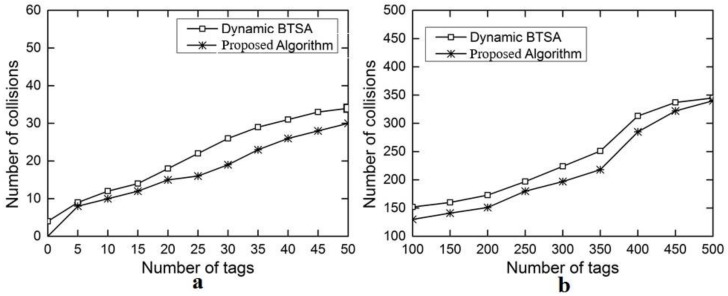
Comparison on the number of collisions between DAF-OS and DBTSA. (**a**) Number of tags in the range 5–50; (**b**) number of tags in the range 100–500.

**Figure 9:**
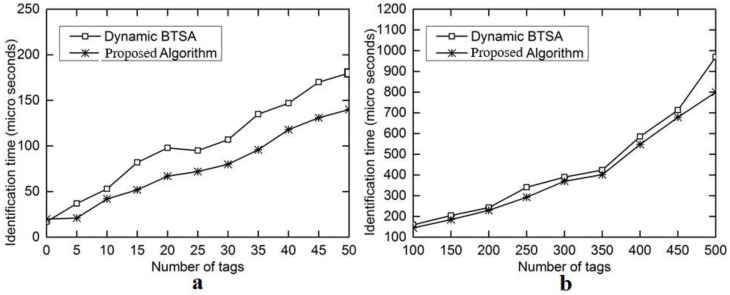
Comparison on the identification time for identifying all tags between DAF-OS and DBTSA. (**a**) Number of tags in the range 5–50; (**b**) number of tags in the range 100–500.

The evaluation results for the number of iterations are shown in Figure 6a with the number of tags ranging from 5 to 50 and in Figure 6b with the number of tags ranging from 100 to 500. This performance gain results from the adoption of the optimal splitting method in DFA-OS. Figure 7a,b and Figure 8a,b show the comparison results on the number of idle slots and the number of collision slots, respectively. As can be seen, the adoption of optimal splitting has resulted in a smaller number of idle slots while dynamic frame adjustment has contributed to a smaller number of collision slots in DAF-OS. Similarly, Figure 9a,b show the comparison results on the identification time between DAF-OS and DBSTA.

**Figure 10:**
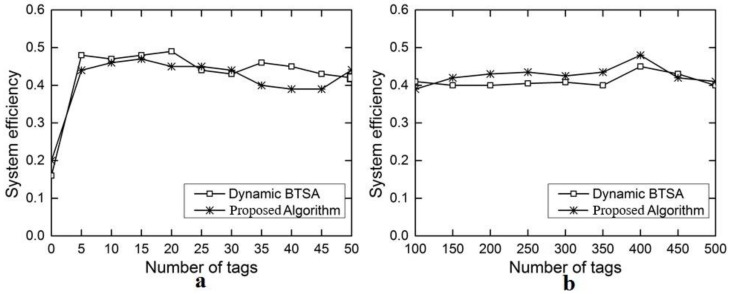
Comparison on system efficiency for identifying all tags between DAF-OS and DBTSA. (**a**) Number of tags in the range 5–50; (**b**) number of tags in the range 100–500.

Figure 10a,b show the comparison results on the system efficiency between DAF-OS and DBSTA. As can be seen, system efficiency fluctuates between 0.4 and 0.5 for both DAF-OS and DBTSA although DAF-OS does show some overall advantage, especially when the number of tags lies within the high range (Figure 10b). System efficiency is defined as the proportion of the total duration of successful slots over all slots during the evaluation period. System efficiency of DAF-OS may become lower than DBTSA at certain points likely due to non-idle slots lasting longer than idle slots, which happens when the duration of a collision slot is shorter than that of a successful slot that is equal to an idle slot.

Table 1 presents the summarized results as perceived from the experiment results. Based on our analysis, we portioned tags into two categories. When number of tags were ranged from 0 to 50, the efficiency of the proposed algorithm (DFA-OS) (0.441) was improved compared to DBTSA algorithm (0.420) in all scenarios exempting the quantity of collisional slots. Whereas, with the number of tags ranged from 100 to 500, the performance of proposed algorithm (0.411) was improved over the DBTSA (0.400) algorithm in all scenarios except the number of collisional slots. However, tags ranging from 100 to 500, the anti-collision algorithm performance was observed to decrease initially. This was due to the fact that the tags were not recognized by the reader in the reading area, and once the tags were identified the efficiency was increased proportionally. The number of collisional slots increased initially due to frame length adjustment by judging the first slot. The positive results yielded by the proposed algorithm led to the improvement in terms of the number of idle slots, number of iterations, and identification time. It can also be inferred from Table 1 that the number of collisions produced were 344 using the modified algorithm compared to 345 using DBTSA, the protocol efficiency achieved less often by the existing protocols such as estimation-based, non-estimation-based, and hybrid-based methods.

## 6. Conclusions

In this paper, a RFID anti-collision algorithm (DFA-OS) was proposed by incorporating dynamic frame adjustment and optimal splitting schemes to improve system efficiency through reducing the number of idle slots as well as the amount of identification time for recognizing all the tags. Experiment showed that the proposed DAF-OS algorithm could achieve maximum efficiency of 0.441 compared to that of 0.420 for the DBTSA algorithm. Moreover, DAF-OS could achieve an overall efficiency gain of 0.032 stably even when the number of tags increases. DAF-OS could also reduce the number of idle slots and iterations, thus reducing the amount of identification time compared to existing protocols including DBTSA although the ratio of collision slots in both algorithms is very close. Through replacing the BT method in DBTSA with the optimal splitting method, DAF-OS could achieve better performance due to reduced size of the idle slot in resolving tag collisions. Our future work will include incorporating optimal splitting with adoptive and splitting BTSA to further improve the efficiency of RFID systems and conducting more experiment and analysis to verify the effectiveness and efficiency of any improvement to RFID tag recognition algorithms.

## Figures and Tables

**Table 1:** Summarized results.

	Proposed Algorithm—Dynamic Binary Tree Slotted ALOHA (DBTSA)				
Number of Tags	Number of Idle Slots	Number of Collisions	Identification Time (Microseconds)	Number of Iterations	System Efficiency
0–50	31–38	30–34	140–180	80–90	0.441–0.420
100–500	392–480	344–345	800–970	850–1050	0.411–0.400
